# The impact of microplastics polystyrene on the microscopic structure of mouse intestine, tight junction genes and gut microbiota

**DOI:** 10.1371/journal.pone.0304686

**Published:** 2024-06-05

**Authors:** Qi-Ling Su, Jiang Wu, Shao-Wen Tan, Xiao-Yun Guo, Ding-Zhe Zou, Kai Kang

**Affiliations:** 1 Department of Veterinary Medicine, College of Coastal Agricultural Sciences, Guangdong Ocean University, Zhanjiang, China; 2 Department of Animal Science, College of Coastal Agricultural Sciences, Guangdong Ocean University, Zhanjiang, China; University of Hawai’i at Manoa, UNITED STATES

## Abstract

Microplastics, which are tiny plastic particles less than 5 mm in diameter, are widely present in the environment, have become a serious threat to aquatic life and human health, potentially causing ecosystem disorders and health problems. The present study aimed to investigate the effects of microplastics, specifically microplastics-polystyrene (MPs-PS), on the structural integrity, gene expression related to tight junctions, and gut microbiota in mice. A total of 24 Kunming mice aged 30 days were randomly assigned into four groups: control male (CM), control female (CF), PS-exposed male (PSM), and PS-exposed female (PSF)(n = 6). There were significant differences in villus height, width, intestinal surface area, and villus height to crypt depth ratio (V/C) between the PS group and the control group(C) (p <0.05). Gene expression analysis demonstrated the downregulation of *Claudin-1*, *Claudin-2*, *Claudin-15*, *and Occludin*, in both duodenum and jejunum of the PS group (p < 0.05). Analysis of microbial species using 16S rRNA sequencing indicated decreased diversity in the PSF group, as well as reduced diversity in the PSM group at various taxonomic levels. Beta diversity analysis showed a significant difference in gut microbiota distribution between the PS-exposed and C groups (R2 = 0.113, p<0.01), with this difference being more pronounced among females exposed to MPs-PS. KEGG analysis revealed enrichment of differential microbiota mainly involved in seven signaling pathways, such as nucleotide metabolism(p<0.05). The relative abundance ratio of transcriptional pathways was significantly increased for the PSF group (p<0.01), while excretory system pathways were for PSM group(p<0.05). Overall findings suggest that MPs-PS exhibit a notable sex-dependent impact on mouse gut microbiota, with a stronger effect observed among females; reduced expression of tight junction genes may be associated with dysbiosis, particularly elevated levels of *Prevotellaceae*.

## 1. Introduction

When plastic enters the environment and degrades, it gradually breaks down into small plastic fragments or particles referred to as microplastics (MPs) with a diameter smaller than 5 mm [1]. Presently, MPs have been detected in many organisms, including the human placenta [2] and blood [3]. Polystyrene (PS) is a polymer synthesized from styrene monomers through free radical polymerization. Smaller particles may be absorbed systemically, and PS is known to accumulate in the liver [4] and intestinal tissues [5] of marine organisms thereby entering the food chain. Many studies have shown that MPs have adverse health effects, including the disruption of lipid metabolism [5], suppression of the respiratory system, inflammatory damage [6], tissue structure damage [7], and the disruption of gene expression [8]. Specifically, PS has been shown to induce dysbiosis of the gut microbiota and disrupt the intestinal barrier of mice [9]. However, it remains to be clarified whether the effect of MPs-PS on the gut microbiota of mice is influenced by sex.

The physical barrier of the intestinal mucosa is a vital component of the intestinal barrier system, in which tight junctions play a crucial role in maintaining its integrity [10]. Tight junctions serve as the primary mode of cell-to-cell adhesion, between intestinal epithelial cells forming highly dynamic structures composed of functional protein molecules such as Claudin proteins (CLDN), Occludin proteins (OCLN), and Zonula Occludin proteins(ZO) [11]. *Occludin* is a protein responsible for preserving the integrity of tight junctions. When disrupted, it can lead to a decrease in the density of tight junctions, resulting in weakened barrier function [12]. Claudin proteins are transmembrane functional proteins contributing to the creation of selective barriers [11]. Among them, *Claudin-1* enhances the tightness of the intestinal barrier, while *Claudin-2*, *Claudin-7* and *Claudin-15* induce increased intestinal permeability [13]. Downregulation of their relative expression levels can result in decreased permeability, affecting the ability of the intestine to absorb nutrients. The ZO protein promotes the formation of tight junction structures and stabilizes the protein skeleton [14]. Decreased expression levels of *Claudin-1* in mice can lead to significant colonic damage, including increased intestinal permeability, mild inflammation, and disruption of the gut microbiota [15]. Similarly, decreased expression levels of *ZO-1* and *Claudin-1* can lead to increased intestinal permeability in BBDP rats prior to the onset of diabetes [16].

At present, the gut microbiota has been utilized as a toxicological target for certain environmental pollutants. The gut microbiota plays a pivotal role in host health by facilitating the breakdown of indigestible components, promoting intestinal homeostasis through resistance against pathogen colonization, and fortifying the intestinal barrier via the provision of energy to the intestinal epithelial cells [17, 18]. It has significant effects on food digestion and absorption, host nutrition, intestinal development, immune response, and physiological regulation [19]. The unbalanced microbiota is prone to inflammation, DNA damage, cancers, and the production of metabolites related to tumor development [20]. The composition and functionality of the gut microbiota are influenced by various factors, including dietary choices, stressors, and antibiotic usage, which subsequently impact the host’s physiological and pathological states [21]. Nan et al. reported neurobehavioral disruption in zebrafish due to changes in the gut microbiota caused by MPs [22]. Similarly, Rawle Daniel J et al. have reported that the persistent retention of MPs in the colon of mice can result in dysbiosis of the gut microbiota, leading to alterations in both the abundance and composition of the microbial community [23]. There is also increasing evidence to suggest that changes in the gut microbiota are closely associated with host immune-inflammatory responses, including conditions such as inflammatory bowel disease [24] and liver dysfunction [25], among others.

The gastrointestinal tract serves as the primary barrier for the entry of MPs into other tissues. a limited number of researchers have investigated alterations in gut microbiota; however, the potential disparities in their effects on males and females remain unclear. This experiment aims to investigate the impacts of commonly encountered MPs on the microscopic structure of the small intestine, expression levels of tight junction proteins, and gut microbiota in mice. determining whether these effects on gut microbiota differ between males and females. These findings will provide valuable and novel data regarding the influence of dietary MPs on mammalian intestinal health and functionality, enabling a more comprehensive assessment of the risks posed by MPs to mammals.

## 2. Materials and methods

### 2.1 Microplastics

Plastic-PS powder was purchased from Meisen Biological Company (Xi’an, China). The microplastics were obtained using a 600-mesh screen (the diameter of PS<25 μm).

### 2.2 Experimental animals and grouping

Mice reached sexual maturity at about 35 days of age, and the intestinal microflora reached a relatively stable state. A total of 24 30-day-old CL-class Kunming mice were randomly and equally divided into control group (C group), and PS group for 4 weeks feeding trial, with six female mice and six male mice in each group. The groups were further labeled as control male group (CM group), control female group (CF group), PS male group (PSM group), and PS female group (PSF group). The C group was provided with homemade feed pellets, The PS group was fed rat cookies at a concentration of 30 g of MS-PS per kilogram for 28 days. During the feeding period, microplastics particles (average at 0.21g/day) infused mouse pellets (average at 7g/day) were quantitatively replaced every 3 days, and mice were free to drink. All the mice were kept in an environmentally controlled room, the temperature was maintained at (25±1)°C, the relative humidity was (65–85)%. The formal experiment was carried out after one week of adaptation. All experimental protocols were approved by the Animal Ethics Committee of Guangdong Ocean University (IACUC No. GDOU-LAE-2020-007).

### 2.3 Mouse feed

The mouse feed was made into cookies by homemade, and the required ingredients and recipe are shown in Table 1. Each kilogram of rodent feed for the PS group was supplemented with 30g of PS MPs.

**Table 1:** Homemade rodent feed composition.

Ingredients	Content(kg)	Ingredients	Content(kg)	Ingredients	Content(kg)
Corn	5.25	Stone powder	0.23	Choline chloride	0.03
Soybean meal	4.5	Ca(H_2_PO_4_)_2_	0.18	Vegetable oil	0.12
Flour	2.1	Salt	0.03	Microminerals	>0.03
Bran	1.5	Multivitamin	0.015	Vitamin C	20
Fish meal	1.05				

### 2.4 Sample collection

After a feeding period of 28 days, mice were euthanized by cervical dislocation. Using sterile surgical forceps, 0.5g of intestinal contents from the ileum, cecum, and colon were collected and transferred into a sterile EP tube, which was then placed on ice. Subsequently, the tube was rapidly frozen in liquid nitrogen and stored at -80°C for subsequent microbiome analysis of the intestinal samples. Tissue samples including uncompressed duodenum, jejunum, and ileum sections measuring 5cm each, were collected and placed in EP tubes containing 4% formaldehyde fixative for structural observation. Additionally, 5mm segments of the duodenum, jejunum, and ileum were collected, washed with physiological saline, flash-frozen in liquid nitrogen, and stored at -80°C for subsequent PCR analysis. The samples used for the preparation of intestinal slices and for PCR analysis are collected from female mice.

### 2.5 Paraffin sectioning and HE staining

Collected intestinal tissues were promptly fixed in formalin and after 12 hours, the formalin was replaced with fresh formalin for an additional 12 hours of fixation. Then, alcohol dehydration with gradient concentration was carried out, followed by wax embedding and sectioning (approximately 4 μm thick). Conventional hematoxylin-eosin staining was performed, and the samples were examined using a microscope (Olympus Corporation, Shinjuku, Tokyo) for image acquisition and analysis.

### 2.6 Gene expression of intestinal tight junction proteins

Total RNA was extracted from flash-frozen intestinal samples using the RNA extraction reagent (G3013-100mL, Servicebio, WuHan, China) following the manufacturer’s instructions RNA quality was assessed using a Thermo Scientific NanoDrop 2000 UV-Vis spectrophotometer (Thermo Fisher Scientific, Waltham, MA, USA) prior to reverse transcription into a cDNA library utilizing the TransGen Reverse Transcription Kit (Golden Biotech Company, Beijing, China). DNA synthesis was conducted with 2 μg of total RNA employing the TransScript One-Step gDNA Removal and cDNA Synthesis SuperMix as per the manufacturer’s protocol (Transotech, Beijing, China). Quantitative Real-Time PCR (qPCR) was performed using TB Green Premix Ex Taq II (Takara Bio Inc., Beijing, China) on a qPCR system (Bio-Rad Laboratories Inc., CA 94547, Hercules California, USA) with the following cycling profile: initial denaturation at 95°C for 5 min followed by 40 cycles of denaturation at 95°C for 10s, annealing at 51°C for 20s, and extension at 72°C for 20s. The data were expressed as relative fold change compared to the average value of the C group. β-actin served as an endogenous control gene. The primers were designed and synthesized by Shanghai Bioengineering Co., Ltd. (Shanghai, China), and are listed in Table 2.

**Table 2:** Primers used in the qPCR reactions.

Gene name	Accession number	Primer sequence	Product size (bp)
*Claudin-1*	NM_016674.4	F:TTCAGGTCTGGCGACATTAG	175
		R:ACAGGAGCAGGAAAGTAGGA	
*Claudin-2*	NM_016675.5	F:TGTCCTCGCTGGCTTGTATTA	396
		R:TGAACTCACTCTTGGCTTTGG	
*Claudin-7*	NM_016887.6	F:CCTGGATTGGTCATCAGATTGTC	175
		R:CGGTACGCAGCTTTGCTTTCA	
*Claudin-15*	NM_021719.4	F:CGTCATCACCACCAACAC	306
		R:GCGTACCACGAGATAGCC	
*Occludin*	XM_029544020.1	F:CCTTCTGCTTCATCGCTTCCTT	164
		R:CGTCGGGTTCACTCCCATTA	
*ZO-1*	NM_001163574.2	F:CAACCAGATGTGGATTTACCC	360
		R:TGATTCTACAATGCGGCGATA	
*β-actin*	NM_007393.5	F:GTACTCTGTGTGGATCGGTGG	124
		R:GGGTGTAAAACGCAGCTCAGT	

### 2.7 Intestine microbiome analysis

The total DNA was extracted from intestinal content samples using the DNeasy PowerSoil Kit (Code No. 47014, QIAGEN, CA, Hamburg, Germany). Quantification was performed using the NanoDrop One spectrophotometer (NanoDrop Technologies, Wilmington, DE) and Qubit 3.0 Fluorometer (Life Technologies, Carlsbad, CA, USA). Specific primers with barcodes were used to amplify the V3-V4 region of the bacterial 16S rRNA gene through PCR. The primer pairs used were 341F (5’-CCTACGGGNGGCWGC’) and 805R (5’- GACTACHVGGGTWTCTAATCC). Library preparation and sequencing were carried out using the VAHTS@ Universal DNA Library Prep Kit for Illumina V3 with VAHTS DNA Adapters set3-set6 for Illumina (Code No. ND607 and N806, NVIDIA, Santa Clara, CA, USA), provided by Benagen Corporation (Wuhan, China), following official tutorials. PCR products underwent purification using magnetic beads while size detection of amplified products was conducted via Qubit analysis and agarose gel electrophoresis methods. After pooling based on read numbers and repairing pooled product ends accordingly, sequencing splices connection took place followed by magnetic bead purification as well as quantification through Qubit analysis. Once passing quality control checks at library level completion stage is reached before high-throughput sequencing is executed using an Illumina NovaSeq 6000 sequencer (Illumina).

### 2.8 Statistical analysis

Using the ImageJ 1.53t software, we measured and calculated four structural parameters using the ruler function at a magnification of 100x for duodenum, jejunum, and ileum samples. These parameters include villus height, villus width, crypt depth, villus surface area, and the ratio of villus height to crypt depth (V/C). The data for each group are presented as mean ± error. The gray value of each gene was obtained by ImageJ 1.53t software, and the relative expression of each group of target genes was calculated by the ratio of the gray value of each target gene in the same group to the gray value of the internal reference gene in the same group. Gene expression data were processed using the 2^-ΔΔ^Ct method in Excel to determine the relative expression levels of target genes in each group. Both structural data and gene expression data were subjected to data validation using t-tests in Excel and GraphPad Prism 9. Intestinal structure as well as intestinal genetic data were mapped using GraphPad Prism 9.

Marker genes were sequenced using the sequence by synthesis (SBS) method on the Illumina sequencing platform to reveal species composition at various taxonomic levels. Silva database and UNITEN database were selected as reference databases. The reference database was trained using the default parameters in QIIME2’s classify-sklearn algorithm, and the Naive Bayes species classifier was constructed, and then the characteristic sequences of each ASVs were annotated to obtain the number of species at different taxonomic levels. Bacterial community alpha diversity was evaluated by calculating Shannon, Chao1, Simpson, ACE and Feature indices. Principal Coordinates Analysis (PCoA) based on operational taxonomic units (OTUs) table was performed using Bray-Curtis algorithm to calculate R2 value and p-value. This analysis aimed to assess differences in species diversity among samples.

Similarity analysis (ANOSIM) with Bray-Curtis algorithm was used to test differences between groups by calculating R-value and p-value. Additionally, PICRUSt2 from Langille Lab was used to analyze gene function in KEGG (Kyoto Encyclopedia of Genes and Genomes) and COG (Clusters of Orthologous Groups of proteins) databases. T-tests were employed to identify significant differences between groups with a significance level set at p<0.05.

The Spearman correlation analysis was conducted to investigate the association between gut microbiota (at the phylum and genus level) and the expression of tight junction protein genes using the OmicStudio tools available at https://www.omicstudio.cn/tool. A significance level of p<0.05 was considered statistically significant, while a trend towards significance was defined as 0.05≤p <0.10.

## 3. Results

### 3.1 Taxonomic analysis of gut microbiota

**Fig 1:**
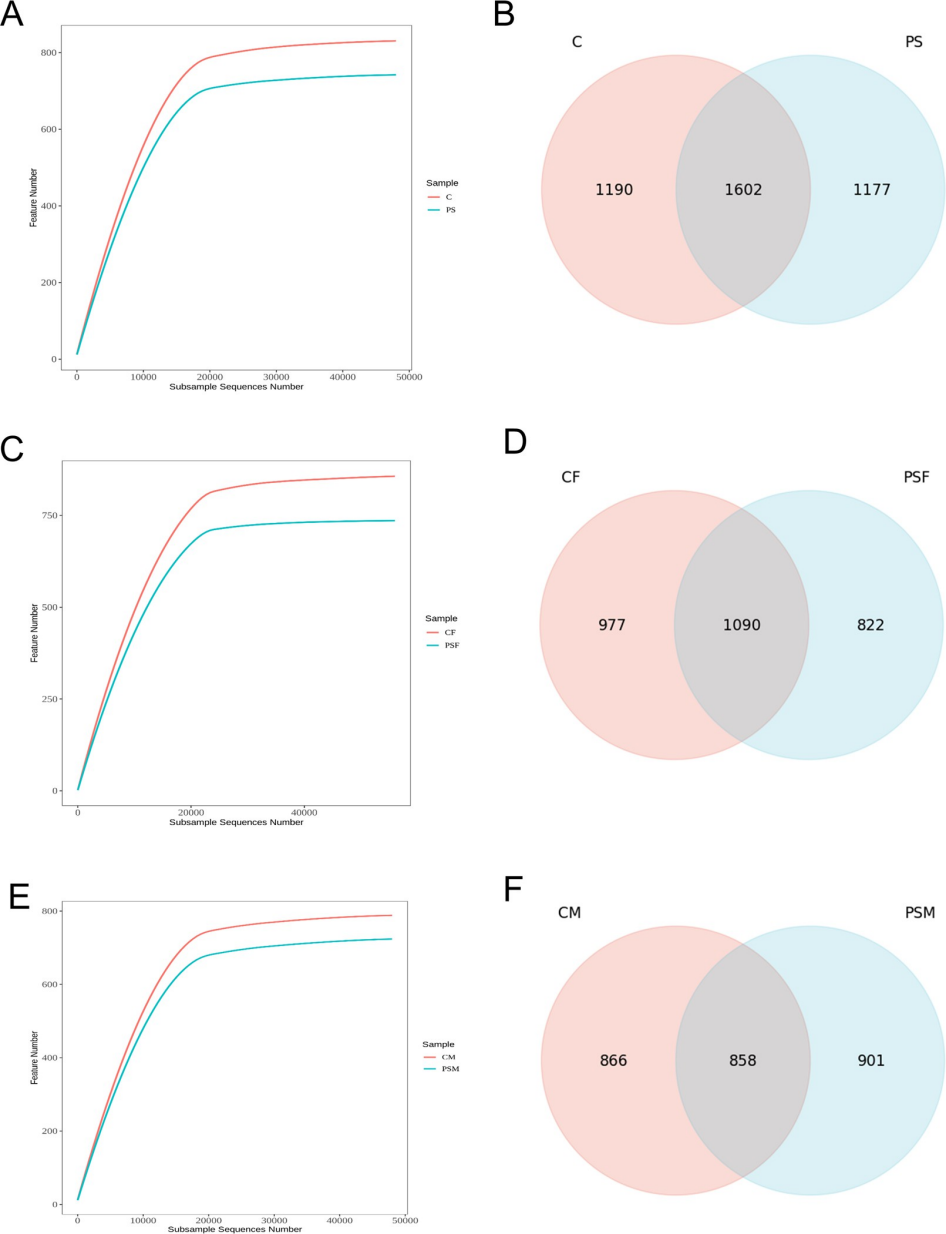
Alpha diversity and common feature analysis of the gut microbial community of mice exposed to microplastics. A. Intergroup dilution curve; B. Common characteristic Venn diagram; C. Intergroup dilution curve of female; D. Common characteristic Venn diagram of females; E. Intergroup dilution curve of male; F. Common characteristic Venn diagram of males.

After 16S rRNA sequencing, a total of 869,750 valid sequences were obtained. The rarefaction curve reached a plateau, indicating sufficient sequencing depth for data analysis (Fig 1A,1C and 1E). The PS group had 1,177 unique features, while the C group had 1,190 unique features with a total of 1,602 shared features between the two groups. Compared to the C group, the PS group showed decreased feature count (Fig 1B). When comparing the CF group to the PSF group, the PSF group displayed 822 unique features whereas the C group had 977 unique features. There were also found to be exactly 858 shared features between these two groups (Fig 1D). In comparison to the CM group, the PSM group showcased 901 unique features while the C group had exactly same number of unique features at 866; there were also found to be exactly same number of shared features at 858 between these two groups (Fig 1F).

The results indicated that exposure to MPs-PS did not significantly impact the overall diversity of microbial taxa at different taxonomic levels (p>0.05) (Table 3). However, compared to the CF group, the PSF group showed a reduction in microbial taxa at various taxonomic levels, with a significant decrease observed at the phylum level (p<0.05) and a highly significant decrease observed at the class level (p<0.01) (Table 4). In contrast, compared to the CM group, the PSM group exhibited an increase in microbial taxa across multiple taxonomic levels including phylum, class, order, family, genus, and species; with a notable increase observed at the order level (p<0.05) (Table 5). The findings suggested that there was gender-specific differences in the impact of microplastics on the gut microbial taxa of mice. However, when conducting a two-way analysis of variance, there were no significant differences among the groups.

**Table 3:** Effect of microplastics on the number of intestinal microbial species in mice (n = 12).

Item	C group	PS group	p-value
Phylum	11.56±0.96	11.33±0.67	0.597
Class	17.11±0.99	16.22±1.31	0.147
Order	36.11±2.08	35.44±2.95	0.608
Family	55.22±3.64	54.22±4.49	0.631
Genus	107.11±4.7	107.78±6.56	0.818
Species	31.78±3.29	32.33±2.36	0.703

**Table 4:** Effect of microplastics on the number of intestinal microbial species in female mice (n = 6).

Item	CF group	PSF group	p-value
Phylum	12.2±0.4	11.4±0.49	0.035
Class	17.8±0.75	15.8±0.75	0.005
Order	37±2	34±3.1	0.142
Family	55.2±3.49	52.2±4.26	0.308
Genus	106.6±5.54	107.2±8.52	0.909
Species	32.4±4.45	32±2.37	0.878

**Table 5:** Effect of microplastics on the number of intestinal microbial species in male mice (n = 6).

Item	CM group	PSM group	p-value
Phylum	10.75±0.83	11.25±0.83	0.488
Class	16.25±0.43	16.75±1.64	0.628
Order	34.75±0.83	37.25±1.3	0.031
Family	54.75±3.27	57±3.54	0.449
Genus	107.25±2.05	108.75±3.56	0.550
Species	30.5±1.12	33.25±2.95	0.182

### 3.2 Gut microbiota species composition

**Fig 2:**
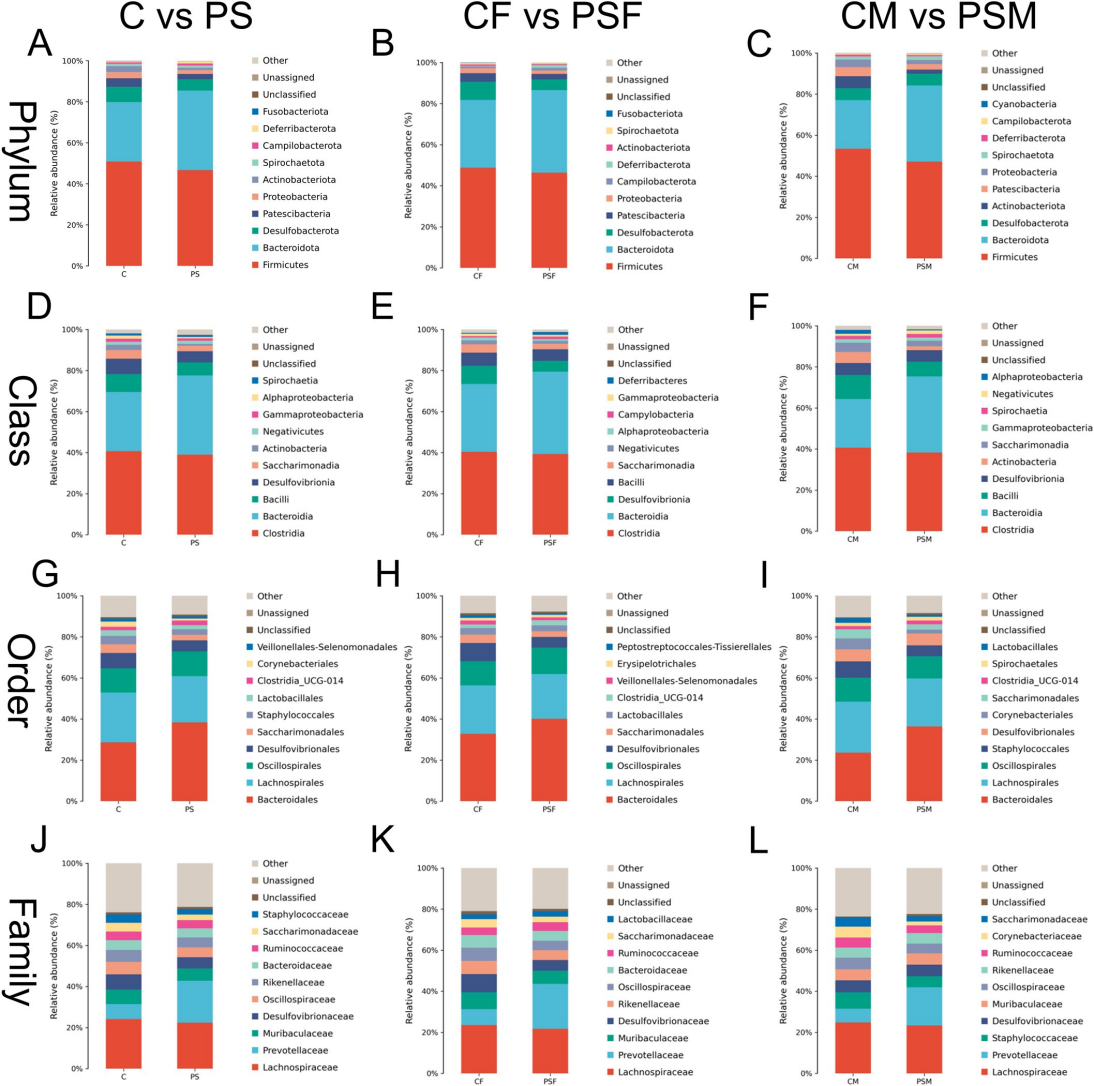
Horizontal species composition bar charts of the species of gut microbiota in mice exposed to microplastics. A. Phylum in all mice; B. Phyla in female mice; C. Phyla in male mice; D. Classes in all mice; E. Classes in female mice; F. Classes in male mice; G. Orders in all mice; H. Orders in female mice; I. Orders in male mice; J. Families in female mice; K. Families in female mice; L. Families in male mice.

Overall, the PS microplastic treatment significantly influenced gut microbiota abundance at various taxonomic levels. Firmicutes and Bacteroidetes were dominant at the phylum level. The relative abundance of Bacteroidetes increased in the PS group, while Desulfobacterota and Patescibacteria decreased. Additionally, there was a decline in the ratio of Firmicutes to Bacteroidetes due to a decrease in the relative abundance of Firmicutes (Fig 2A). At the class level, Clostridia and Bacteroidia were dominant. Compared to the C group, there was an increase in the relative abundance of Bacteroidia within the PS group, while Bacilli and Desulfovibrionia exhibited decreased abundances (Fig 2D). Moving on to the order level, Bacteroidales and Desulfovibrionales held dominance. The PS group showed an increase in relative abundance for Bacteroidales but a decrease for Desulfovibrionales (Fig 2G). Lastly, family-level analysis revealed *Lachnospiraceae* and *Prevotellaceae* as dominant families. Within the PS group, there was an increase in relative abundance for *Prevotellaceae* family; however, *Lachnospiraceae* along with *Muribaculaceae* exhibited decreased abundances alongside *Desulfovibrionales* (Fig 2J).

In female mice exposed to PS, the dominant phyla were Firmicutes and Bacteroidetes, with an increased relative abundance of Bacteroidetes in the PSF group. Conversely, there was a significant decrease in Desulfobacterota and Patescibacteria.(Fig 2B) At the class level, Clostridia and Bacteroidia were identified as dominant classes, with an increased relative abundance of Bacteroidia and a decreased presence of Desulfovibrionia in the PSF group. Additionally, Bacilli showed a notable decline. (Fig 2E). The orders Bacteroidales and Desulfovibrionales were determined to be dominant; however, compared to the C group, there was an increased relative abundance of Bacteroidales while Desulfovibrionales showed a decrease in the PSF group. (Fig 2H). At the family level, *Lachnospiraceae* and *Prevotellaceae* demonstrated dominance (Fig 2K). Furthermore, when comparing with C group, it was observed that *Muribaculaceae* and *Desulfovibrionaceae* had reduced relative abundances whereas *Prevotellaceae* exhibited an increase.

In male mice exposed to PS, the dominant phyla in their gut microbiota were Firmicutes and Bacteroidetes (Fig 2C). The relative abundance of Bacteroidetes decreased while that of Firmicutes and Proteobacteria increased in the PSM group. At the class level, Clostridia and Bacteroidia were identified as dominant classes, with a decrease in the relative abundance of Bacteroidia and an increase in Bacilli observed in the PSM group (Fig 2F). The orders Rikenellales and Bacteroidales were found to be dominant, specifically with a decrease observed for Bacteroidales in the PSM group (Fig 2I). *Lachnospirales* and *Prevotellaceae* showed dominance at the family level (Fig 2L). Notably, there was an increase in *Staphylococcales*’ relative abundance while a decrease was observed for *Prevotellaceae* compared to the C group.

Overall, Fusobacteriota exhibited the most significant decrease in relative abundance at the phylum level in the PS group, while Actinobacteriota experienced the largest decline in relative abundance in the PSM group. In the PSF group, Fusobacteriota showed a substantial decline, contributing to its decreased presence compared to the PS group (Fig 2B). At the class level, Actinobacteria displayed a notable reduction within the PS group, whereas Deferribacteres demonstrated an increase within the PSF group. The PSM group showed considerable decreases in Alphaproteobacteria and Actinobacteria, which primarily contributed to overall declines observed within Actinobacteria at phylum level among mice exposed to microplastics (Fig 2F). At order level, Corynebacteriales experienced a significant decrease in relative abundance within the PS group. *Prevotellaceae* exhibited a prominent increase at family level within this same group. Desulfovibrionales had noteworthy decreases at both order and family levels within the PSF group; meanwhile Corynebacteriales experienced major declines within PSM groups (Fig 2I and 2L). These findings indicated that microplastics had varying effects on gut microbiota abundance between male and female mice.

### 3.3 Gut microbial diversity

The impact of PS on gut microbiota abundance and diversity in mice was assessed. Alpha diversity analysis (Table 6) showed a decreasing trend for Chao-1, ACE, Shannon, and Simpson indices under PS exposure; however, these changes were not statistically significant (P>0.1). Both the PSF and PSM groups also had non-significant decreases in all indices compared to the CF and CM group, respectively (P>0.1) (Tables 7 and 8).

**Table 6:** Effects of microplastics on the alpha diversity of the intestinal microbiome in mice (n = 12).

Items	C group	PS group	p-value
Feature	802.78±95.68	746.44±88.92	0.240
ACE	810.11±97.53	753.51±91.54	0.249
Chao1	808.8±97.07	752.35±91.19	0.248
Shannon	7.7±0.52	7.41±0.55	0.290
Simpson	0.98±0.01	0.98±0.02	0.261

**Table 7:** Effects of microplastics on α diversity of intestinal microbes in female mice (n = 6).

Items	CF group	PS group	p-value
Feature	808.6±80.32	737.8±99.89	0.301
ACE	812.58±80.87	740.58±102.04	0.301
Chao1	811.53±80.57	739.34±101.41	0.297
Shannon	7.81±0.14	7.35±0.65	0.208
Simpson	0.99±0	0.97±0.02	0.234

**Table 8:** Effects of microplastics on α diversity of intestinal microbes in male mice (n = 6).

Items	CM group	PS group	p-value
Feature	802.78±95.68	746.44±88.92	0.667
ACE	810.11±97.53	753.51±91.54	0.642
Chao1	808.8±97.07	752.35±91.19	0.658
Shannon	7.7±0.52	7.41±0.55	0.836
Simpson	0.98±0.01	0.98±0.02	0.834

**Fig 3:**
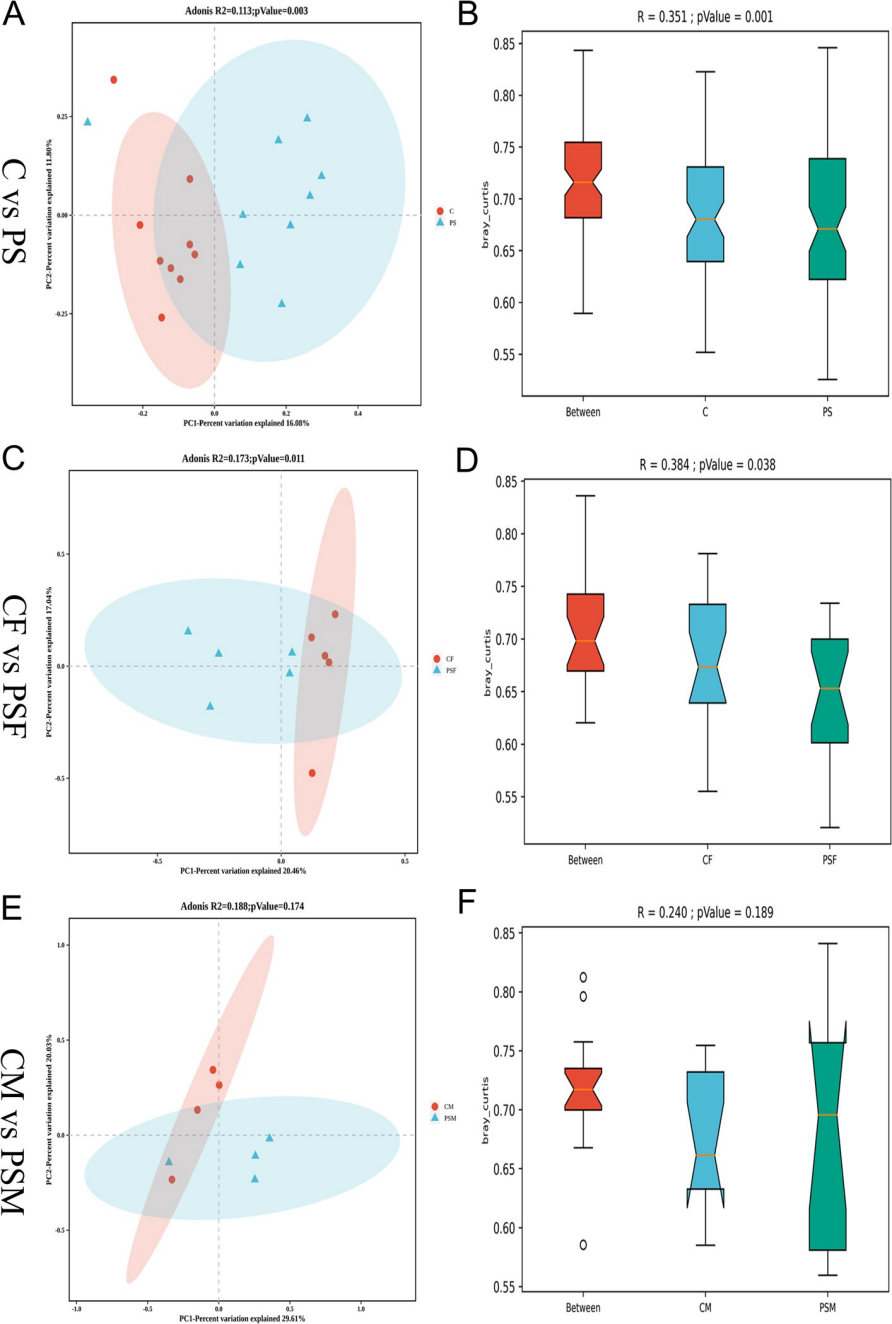
Beta diversity analysis of gut microbial diversity in ice exposed to microplastics. A. PCoA graph based on Bray Curtis algorithm; B. Anosim graph based on Bray Curtis algorithm; C. PCoA graph of female mice; D. Anosim graph of female mice; E. PCoA graph in male mice; F. Anosim graph in male mice.

The beta diversity analysis compared species diversity among different microbial samples. Principal coordinate analysis (PCoA) revealed a significant inter-group difference in gut microbiota distribution between the PS group and the C group (R2 = 0.113, P = 0.003) (Fig 3A). Additionally, while intra-group differences in gut microbiota were relatively small within the PS group compared to the C group, highly significant differences were observed between the PS group and the C group (R = 0.351, p = 0.001) (Fig 3B). These findings suggest that microplastic contamination from PS significantly impacts mice’s gut microbiota composition.

The PSF group showed strong clustering within the group and significant differences compared to the CF group (R2 = 0.173, P = 0.011) (Fig 3C). ANOSIM analysis further revealed that while there were minor variations in gut microbiota composition within the PSF group, the differences between groups were statistically significant (R = 0.384, p = 0.038) (Fig 3D). There was no notable distinction between the PSM group and CM group (R2 = 0.173, P = 0.174) (Fig 3E). However, within the PSM group, there were considerable dissimilarities in gut microbiota composition compared to the CM group; yet no significant differences were found between the PSM and C groups (R = 0.240, p = 0.189) (Fig 3F), suggesting a more pronounced detrimental impact of PS microplastic contamination on female mice’s gut microbiota.

### 3.4 Functional prediction analysis of gut microbiota

**Fig 4:**
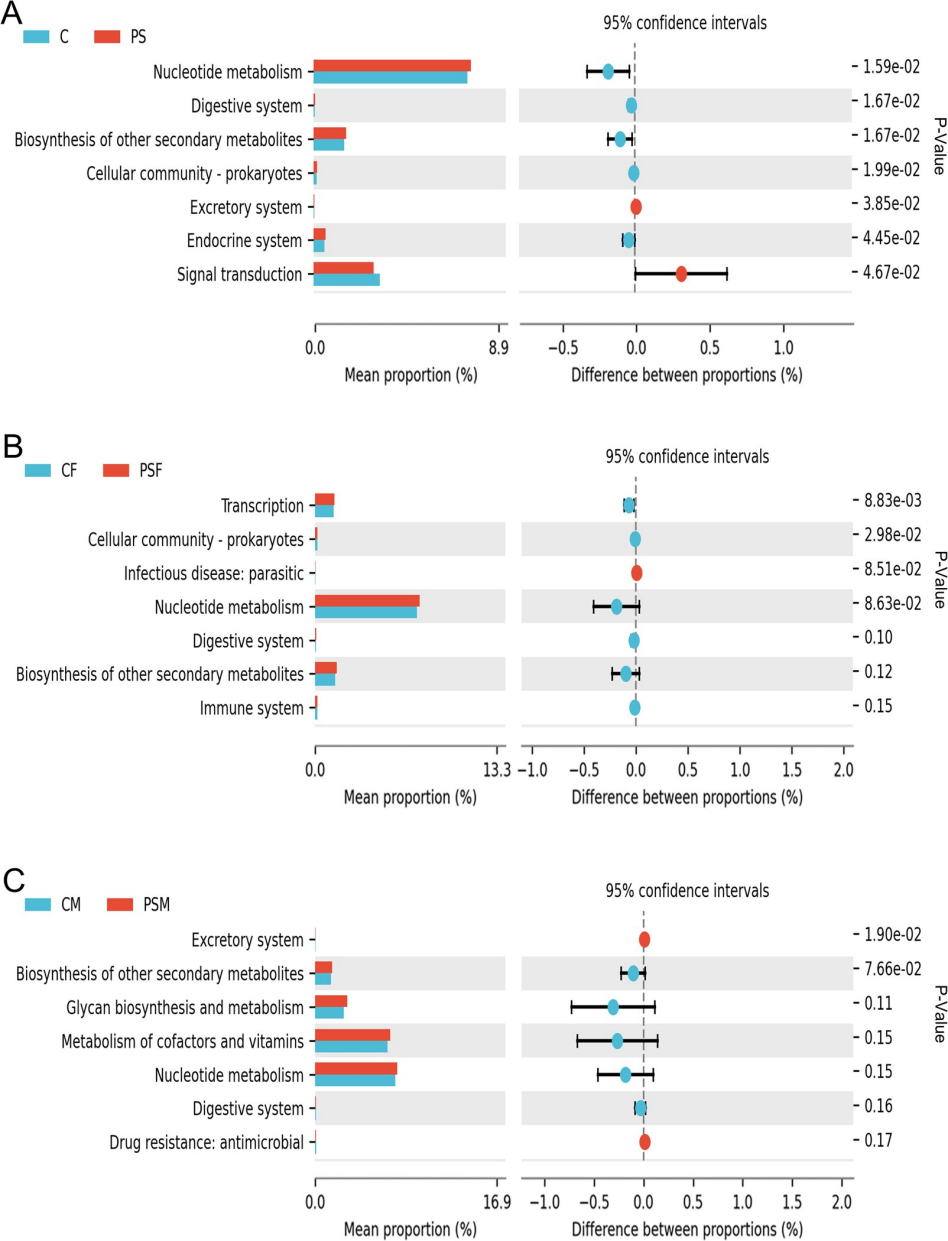
KEGG functional difference analysis of functional genes and their associated metabolic pathways in mice exposed to microplastics. A. KEGG Level 2 functional differences histogram; B. KEGG Level 2 functional differences histogram of females; C. KEGG Level 2 functional differences histogram of males.

In the KEGG functional analysis, the PS group showed a significant increase in relative abundance of nucleotide metabolism, digestive system, biosynthesis of other secondary metabolites, cellular community (prokaryotes), and endocrine system pathways compared to the C group (p<0.05). However, there was a significant decrease in activity observed for excretory system and signal transduction pathways (p<0.05) (Fig 4A). The PSF group had a highly significant increase in transcription pathways’ relative abundance (p<0.01) and a significant increase in cellular community (prokaryotes) pathway’s relative abundance compared to the CF group (p<0.05) (Fig 4B). Additionally, PSM group displayed a notable decrease specifically within the excretory system pathway when compared to the CM group(p<0.05) (Fig 4C).

**Fig 5:**
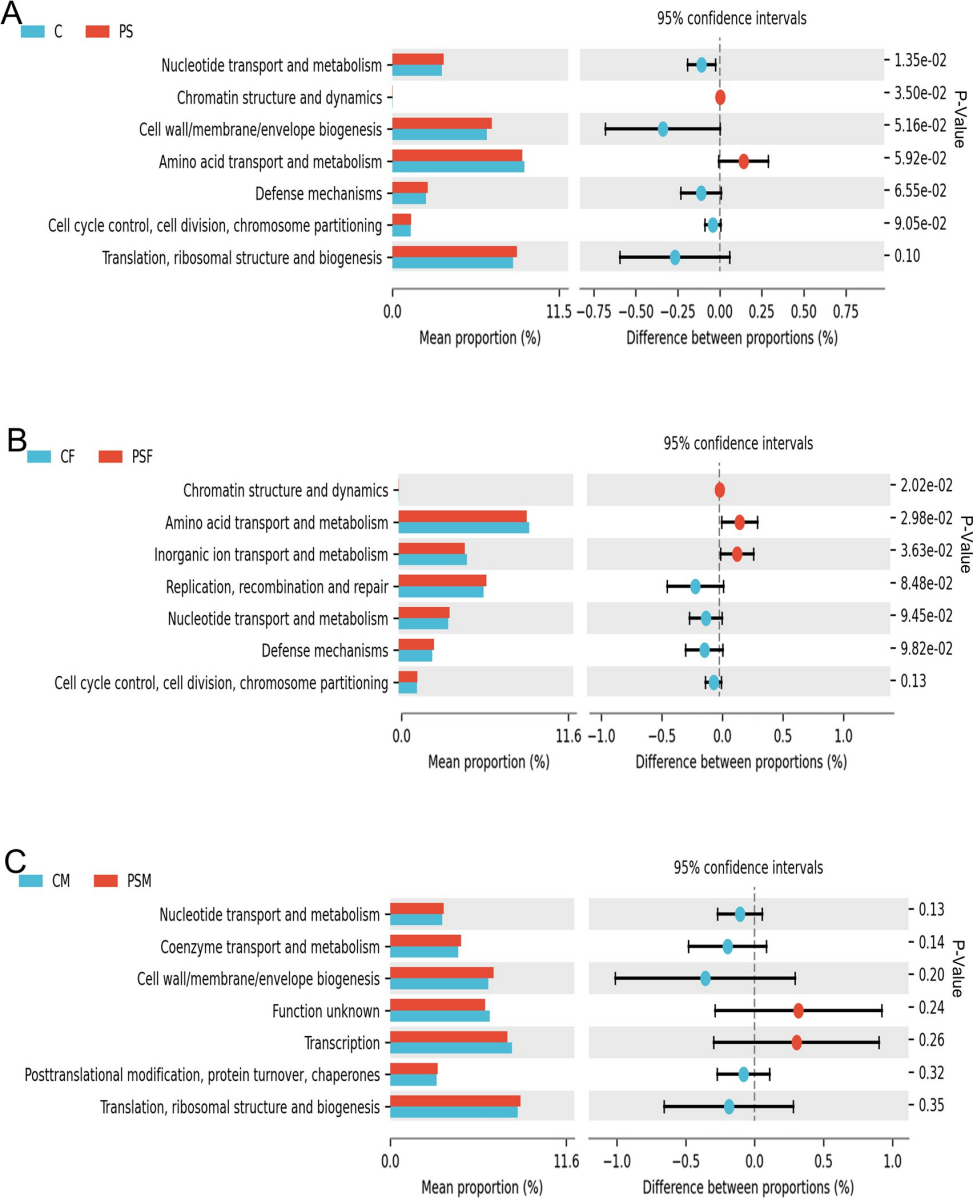
COG functional difference analysis of functional genes and their associated metabolic pathways in mice exposed to microplastics. A. COG functional difference bar chart; B. COG functional difference bar chart for females; C. COG functional difference bar chart for males.

The COG functional composition analysis showed a significant increase (p<0.05) in nucleotide transport and metabolism genes in the PS group, while chromatin structure and dynamics genes were significantly decreased (p<0.05) (Fig 5A). In the PSF group, there was a significant decrease (p<0.05) observed in chromatin structure and dynamics, amino acid transport and metabolism, as well as inorganic ion transport and metabolism genes (Fig 5B). No significant changes were found in the functional genes analyzed by COG for the PSM group (Fig 5C).

### 3.5 Impact of MPs-PS on small intestine structure of female mice

**Fig 6:**
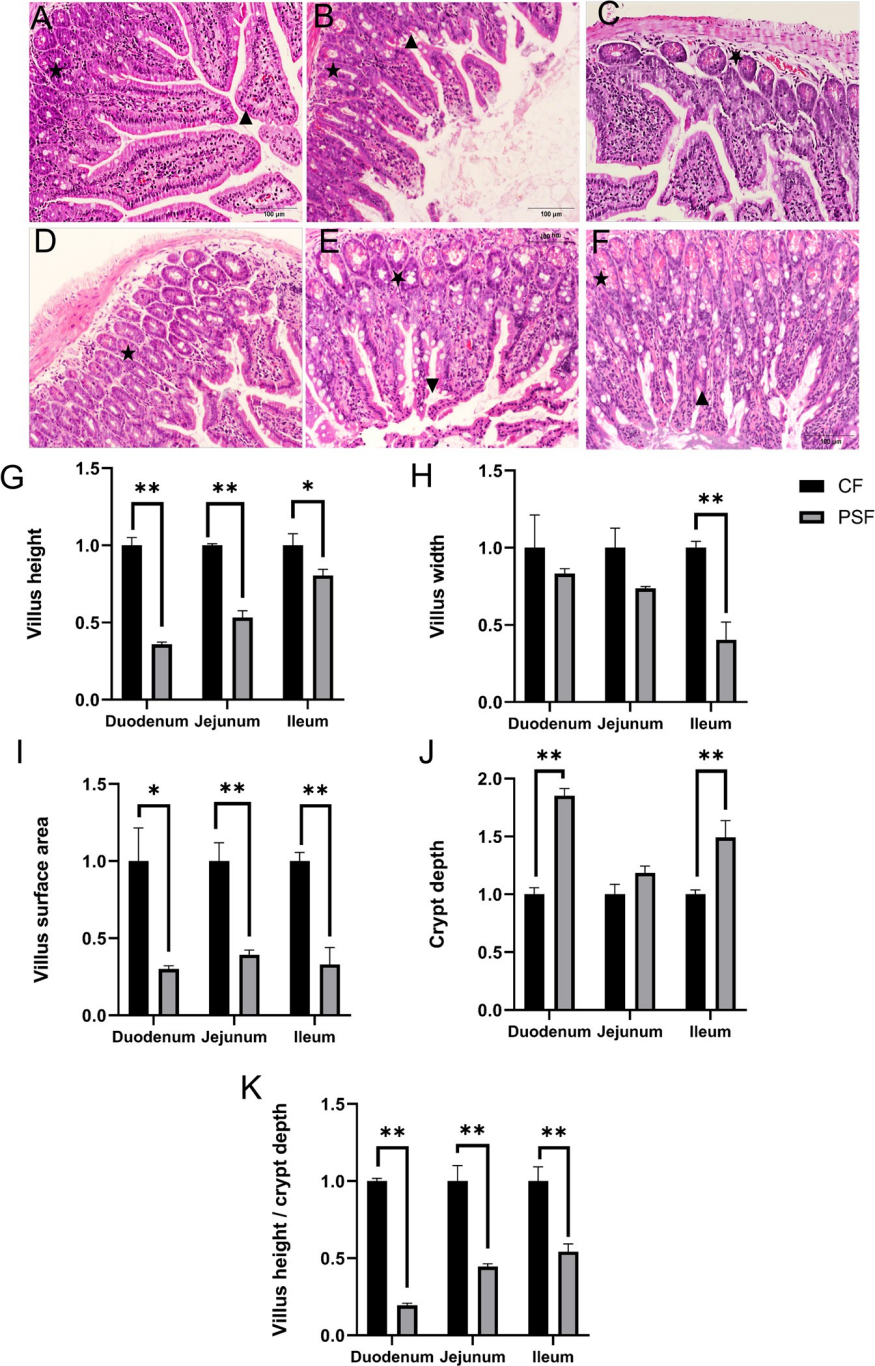
Intestine section and microstructure analysis of female mice exposed to microplastics. A. Duodenum of CF group (100×); B. Duodenum of PSF group (100×); C. Jejunum of CF group (100×); D. Jejunum of PSF group (100×); E. Ileum of CF group (100×); F. Ileum of PSF group (100×); G. Villus height; H. Duodenal villus depth; I. Duodenal villus surface area; J. Duodenal crypt depth; K. Duodenal villus height/crypt depth V/C value; *: p<0.05; **: p<0.01.

The evaluation of the HE-stained sections showed that MPs-PS had comparable detrimental effects on the microstructure of different segments of the mouse small intestine. These effects included disruption of villus structure (Fig 6B▲ and 6F▲), decreased villus height, reduced villus surface area, increased crypt depth (Fig 6B★, 6D★ and 6F★), and a decline in the V/C ratio. Specifically, there was a statistically significant reduction in villus height observed in both duodenum and jejunum compared to the CF group (p<0.01), while it exhibited a significantly lower level (p<0.05) in ileum (Fig 6G). Moreover, there was a highly significant decrease in villi width observed in ileum compared to the CF group (p<0.01) (Fig 6H). Additionally, there were highly significant reductions noted in jejunum and ileum regarding villi surface area when compared to the CF group (p<0.01), with a significant decrease seen in duodenum as well (p<0.05) (Fig 6I). Furthermore, an increase in crypt depth was highly significant for both duodenum and ileum compared to the CF group(p<0.01) (Fig 6J), whereas a significantly decreased V/C ratio was observed for all three segments: duodenum, jejunum, and ileum when compared to the CF group (p <0.01) (Fig 6K). These findings indicate that PS had more pronounced adverse effects on microstructural integrity within duodenum than those evident within jejunum or ileum.

### 3.6 Impact of MPs-PS on the expression of tight junction protein genes of female mice

**Fig 7:**
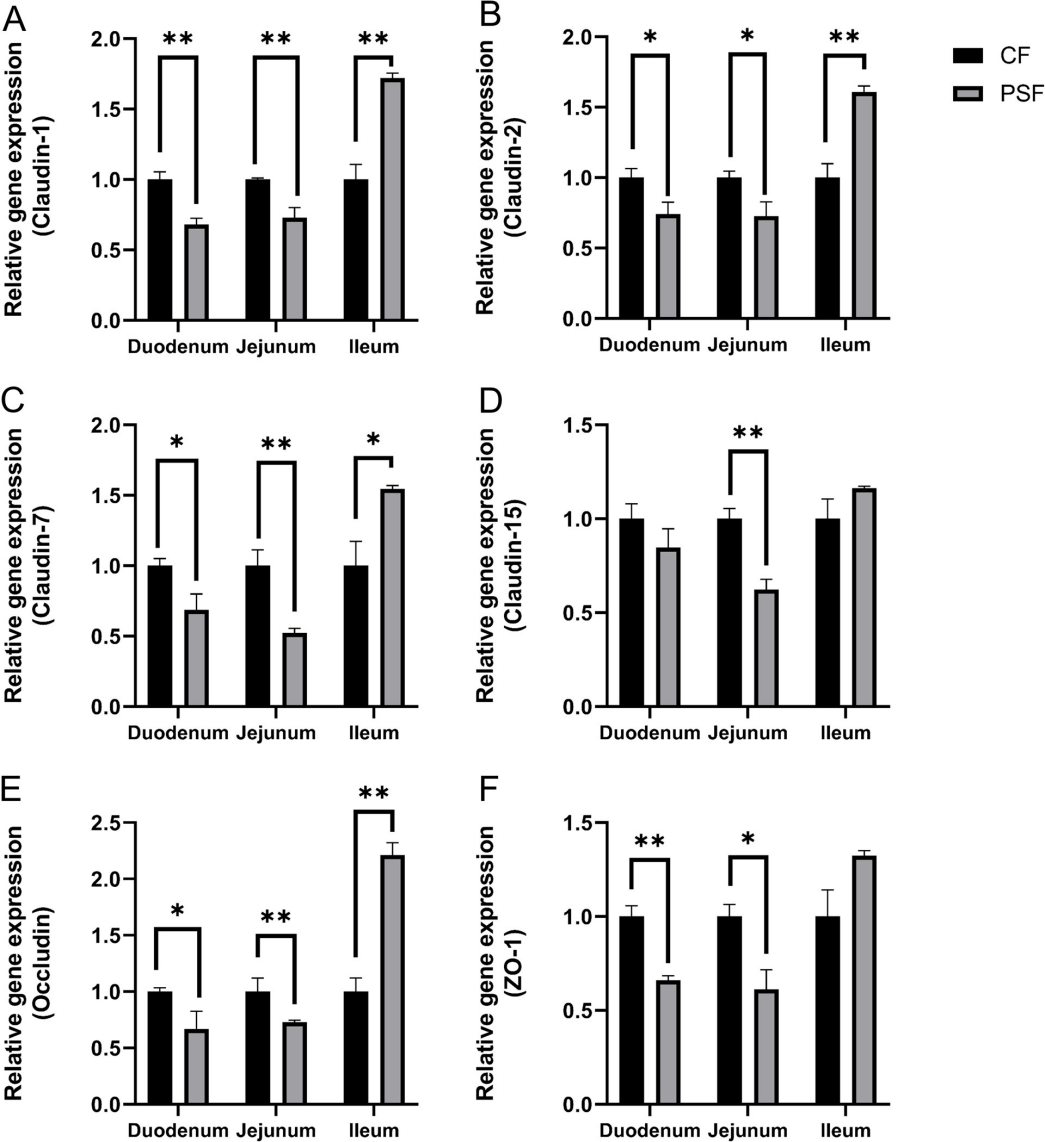
The relative expression levels of intestinal tight junction genes in female mice exposed to microplastics. A. Claudin-1; B. Claudin-2; C. Claudin-7; D. Claudin-15; E. Occludin; F. ZO-1; *: p<0.05; **: p<0.01.

In the PSF group, relative expression levels of *Claudin-1*, *Claudin-2*, *Claudin-7*, *Claudin-15*, *Occludin* and *ZO-1* genes were downregulated in the duodenum and jejunum but upregulated in the ileum compared to the CF group. Specifically, compared to the CF group, there was a highly significant downregulation (p<0.01) of *Claudin-1* expression in both duodenum and jejunum while its expression was highly significantly upregulated (p<0.01) in ileum (Fig 7A). Similarly, there was a significant downregulation (p<0.05) of *Claudin-2* expression in both duodenum and jejunum while its expression was highly significantly upregulated (p<0.01) in ileum (Fig 7B). The relative expression level of *Claudin-7* showed significant downregulation (p<0.05) in duodenum and jejunum but highly significant upregulation (p<0.01) in ileum (Fig 7C); whereas that of *Claudin-15* showed high significance downregulation only in jejunal tissue samples(p<0.01) (Fig 7D). Furthermore, *Occludin*’s relative gene expressions were found to be significantly decreased (p<0.05) and highly significantly increased(p<0.01) respectively for duodenal and ileal tissues, while it remained unchanged for Jejunal tissues (Fig 7E). In the process of conducting two-way analysis of variance, the expression of *Claudin-2*、*Claudin-7*、and *Occludin* is not significant (P<0.01). Finally, *ZO-1*’s relative gene expressions were found to be highly significantly decreased (p<0.01) for duodenal tissues and significantly decreased (p<0.05) for jejunal tissues (Fig 7F). In the process of conducting two-way analysis of variance, the expression of *Occludin* in the duodenum and ileum is significant (P<0.05), while the expression of *ZO-1* in the ileum is highly significant (P<0.01).

### 3.7 Correlation between gut microbiome and tight junction protein genes expression of female mice

**Fig 8:**
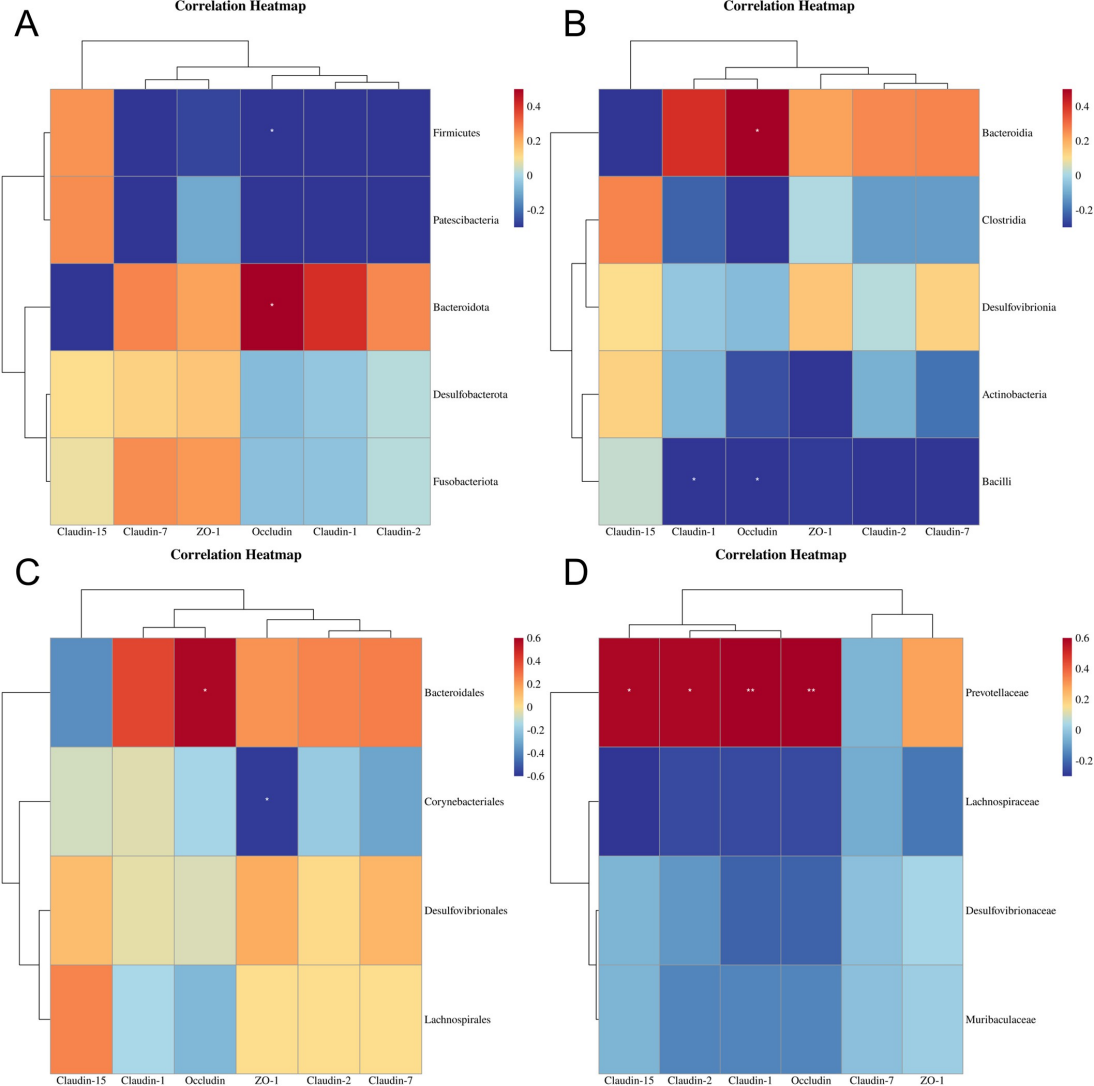
Spearman correlation analysis between the Gut microbials and tight junction protein genes expression of female mice exposed to microplastics at the phylum(A), class(B), order(C) and family(D) level, respectively (n = 6). *: p<0.05; **: p<0.01.

The results of Spearman correlation analysis (Fig 8) showed that at the phylum level, *Bacteroidota* had a positive association with *Occludin* expression (p < 0.05), while *Firmicutes* had a negative correlation with *Occludin* expression (p < 0.05). At the class level, *Bacteroidia* was positively related to *Occludin* expression (p < 0.05), whereas Bacilli had a negative correlation with Claudin-1 and *Occludin* expression (p < 0.05). At the order level, *Bacteroidales* displayed a positive association with *Occludin* expression (p < 0.05), while *Corynebacteriales* showed a negative correlation with *ZO-1* expression (p < 0.05). Lastly, at the family level, *Prevotellaceae* exhibited positive associations with *Claudin-1*5 and *Claudin-2*(p < 0.05), as well as *Claudin-1* and *Occludin* expression (p < 0.01).

## 4. Discussion

The intestine serves as the primary organ responsible for digestion and absorption in animals, with its microstructure playing a pivotal role in facilitating efficient nutrient uptake. Additionally, the intestinal mucosa acts as a crucial barrier against infections. In this context, maintaining a stable expression of tight junction proteins is imperative to uphold the physical integrity of the intestinal mucosa, thereby promoting effective energy and nutrient extraction from food, preventing infections, regulating immune function, and reinforcing the biochemical barrier within the intestine. Consequently, alterations in the distinctive structure of the small intestine closely correlate with changes in nutrient absorption within an organism’s body.

This study demonstrated that PS MPs significantly affect the small intestine in mice, causing a reduction in villus height, disruption of villus integrity, decreased villus surface area, increased crypt depth, and an unclear crypt structure. Additionally, exposure to PS also resulted in a decreased ratio of villus height to crypt depth. These findings align with Pedà et al.’s discovery that PVC MPs caused shortening and swelling of intestinal villi in sea bass, leading to structural fusion and apical rupture [26]. The alterations in crypt structure may reduce mature cell production from the base of the crypt, resulting in diminished mucous secretion and absorption capacity. Disruption of the crypt structure leads to decreased mucus secretion by goblet cells which play a protective role for the intestine. This weakened barrier function makes it easier for pathogenic bacteria to penetrate the intestinal epithelium [27]. Similarly, the decreased ratio of villus height to crypt depth indicates an incomplete morphological structure of the intestine. Moreover, reduced small intestinal epithelial cells and impaired maturity can further compromise the synthesis and secretion rates of digestive enzymes, thereby weakening absorption capacity [28]. These observations provide evidence that MPs disrupt intestinal structure impair absorption capacity, and compromise defense mechanisms, leading to digestive dysfunction and compromised barrier functions within mouse small intestines.

Specifically, the expression of normal tight junction protein genes is closely linked to the integrity of intestinal epithelial cell connections and the intestinal barrier system. Gene expression analysis revealed a decrease in the relative expression levels of these genes in both the duodenum and jejunum. This downregulation of Claudin family genes in these regions reduces intestinal permeability and disrupts the structure of the intestinal barrier, leading to an imbalance in the immune system and initiating inflammatory responses. Previous studies have shown that reduced expression levels of *Claudin-1* and *ZO-1* in mice’s colon can cause significant damage [15]. In our study, we observed a downregulation in relative expression levels of tight junction proteins *Claudin-1* and *ZO-1* within the duodenum and jejunum of experimental mice, resulting in decreased stability within their tight junction protein skeleton as well as reduced tightness, ultimately causing intestinal damage. The research by Jingshen Zhuang indicates that the combined exposure to PS and PVC leads to a decrease in the expression of *ZO-1* [29]. Conversely, our experiment demonstrated an upregulation in relative expression levels for members of the Claudin family, *Occludin*, and *ZO-1* within the ileum region; this increase contributed to enhanced intestinal permeability. The increased expression level of *Occludin* potentially strengthens tight junction structures while enhancing barrier function-potentially aiding repair processes following exposure to MPs.

In addition to maintaining intestinal structural integrity, a well-preserved and balanced microbial community is crucial for optimal health. Disruption of the gut microbiota can cause inflammation and damage to the intestinal mucosal barrier, leading to intestinal inflammation. The study findings indicate that PS had no significant impact on the diversity of the intestine’s microorganisms. However, analysis of beta diversity revealed a disturbance in the gut microbial community among mice exposed to PS. Wang’s study demonstrated that Ms-PS caused substantial changes in both alpha and beta diversity analyses of mouse gut microbiota structure [30]. This suggests that exposure to microplastics, such as PS, significantly influences not only the composition and structure but also potentially impacts species richness and evenness within mouse gut microbiota.

Additionally, no significant sex disparities were observed in the impact of PS on gut microbiota species richness and diversity in mice based on alpha diversity analysis. Additionally, no significant sex disparities were observed in the impact of PS on gut microbiota species richness and diversity in mice based on alpha diversity analysis [31]. Jing observed no significant changes in beta diversity between the male mice exposed to PS and the untreated C group [32]. However, female mice exposed to PS exhibited notable dissimilarities in microbiota composition according to both PCoA and ANOSIM analyses, while males showed no such differences. This suggests a sexual dimorphism in the influence of PS microplastics on gut microbiota composition, with a more pronounced effect seen in females.

Two additional studies have demonstrated a reduction in the relative abundance of the phylum Firmicutes in the gut as a result of exposure to PS [9, 33]. This decrease is potentially linked to impaired intestinal anti-inflammatory effects and compromised intestinal barrier function [34]. Short-chain fatty acids serve as crucial metabolites for the gut microbiota, playing a vital role in maintaining the integrity of the intestinal epithelial barrier and alleviating inflammatory bowel disease. Firmicutes can enhance the host’s absorption of short-chain fatty acids. Therefore, the decline in relative abundance of Firmicutes in the PS group indicates reduced absorption of short-chain fatty acids, leading to decreased energy absorption within the body and weakened efficacy of short-chain fatty acids in mitigating inflammatory bowel disease. In comparison to Group C, female mice exhibited a more pronounced decrease in relative abundance of Firmicutes, consistent with experimental findings by Siyue et al. [15]. Conversely, there was an observed increase in the relative abundance of *Prevotellaceae* within the PS group, suggesting compensatory mechanisms within the intestine by increasing another microbial taxon to maintain overall intestinal functionality. *Prevotellaceae* has been associated with elevated levels of short-chain fatty acids within the body [35]. *Prevotellaceae* genus has been demonstrated to be associated with male reproductive disorders [36, 37].This further demonstrates that there exists a dynamic balance among gut microbiota for sustaining normal physiological functions within hosts.

The Bacteroidetes play a crucial role in digestion and nutrient absorption [38]. In this experiment, Bacteroidetes dominated at the phylum, class, and order levels in the mouse gut. Compared to the C group, there was an increase in Bacteroidetes’ relative abundance in the PS group, possibly due to elevated oxidative stress caused by exposure to microplastics [39]. Some researchers have found that the relative abundance of the Bacteroides genus in the bodies of workers residing in areas with high microplastic exposure is significantly lower compared to those in areas with low microplastic exposure [40], contrary to the results of this study. Under PS microplastic pollution conditions, organisms experience heightened levels of oxidative stress. It is worth noting that the increased relative abundance of Bacteroidetes resulting from PS pollution alters their ratio with non-Bacteroidetes phyla, which can impact carbohydrate metabolism and fat metabolism rates in mice [41]. Furthermore, studies suggest that an increase in Bacteroidetes’ relative abundance can enhance metabolic byproducts triggering an inflammatory response leading to disruption of intestinal mucosal barrier function [42]. Additionally, various bacterial groups within Bacteroidetes release toxic substances during protein degradation. These metabolic byproducts can activate inflammatory signaling pathways and affect intestinal barrier function while exacerbating inflammatory responses [43]. Sequencing results also revealed a decrease in Firmicutes-to-Bacteroidetes ratio within the PS group consistent with Sun et al.’s findings [43]. However, other researchers have reported no change or even an increase in Firmicutes-to-Bacteroidetes ratio with PS exposure [44], whereas changes in this ratio are considered markers of gut dysbiosis [45] since both Firmicutes and Bacteroidetes participate in carbohydrate degradation and fermentation processes; thus a decreased Firmicutes-to-Bacteroidetes ratio suggests weakened regulation of the intestinal environment.

The *Lachnospirales*, a member of the Firmicutes phylum, includes species capable of producing short-chain fatty acids and possessing anti-inflammatory and immunomodulatory functions [46–49]. In this study, a reduction in the abundance of *Lachnospirales* within the gut microbiota of mice in the PS group was observed at both the order and family levels. Surana et al. found a negative correlation between the *Lachnospirales* family and intestinal inflammation [50]. Therefore, it can be inferred that PS MPs reduce endogenous production of short-chain fatty acids, compromise intestinal epithelial cell barrier function, attenuate anti-inflammatory effects, and increase susceptibility to intestinal inflammation.

The KEGG analysis revealed a significant decrease in the relative abundance of specific signaling pathways closely associated with environmental information processing due to PS. This suggests that PS MPs may impair the mouse’s ability to process environmental information. The gut microbiota of the PS group exhibited an increased relative abundance of Prevotella, which plays a role in protein and carbohydrate breakdown, indicating a strong association with the notable increase in the digestive system pathway. COG analysis demonstrated significant alterations in functional genes related to metabolic processes in mice exposed to PS MPs, with variations observed between males and females. Notably, there was a significant downregulation of genes associated with chromatin structure and dynamics, amino acid transport and metabolism, as well as inorganic ion transport and metabolism specifically within the PSF group, while no significant changes were observed within the PSM group. These functional genes are intricately linked to metabolism. The decrease in Firmicutes [17] and increase in Bacteroidetes [51] among female mice is more pronounced compared to male mice. The phyla Bacteroidetes and Firmicutes are closely connected to mouse metabolism, which could explain the decline in metabolic levels among female mice. Zhao Nan indicates a close association between exposure to microplastics (MP) and significant changes in the gut microbiota and metabolic profile of rats [52]. Intestinal bacteria and their metabolites contribute to enhancing the integrity of the epithelial barrier. Therefore, a decrease in the diversity of intestinal bacteria and a reduction in metabolic activity often signify a weakened ability of the host to resist the invasion of pathogens and other threats.

The interaction between gut microbiota and tight junctions in mice exposed to microplastics was investigated using Spearman correlation analysis. Notably, the expression of *Occludin*, a key component of tight junctions, showed significant correlations: at the phylum level, Bacteroidota had a positive association (p<0.05), while Firmicutes displayed a negative correlation with its expression (p<0.05); at the class level, Bacteroidia demonstrated a positive relationship (p<0.05), whereas Bacilli and *Claudin-1* were negatively correlated with it (p<0.05); at the order level, Bacteroidales exhibited a positive association with its expression (p<0.05); at the family level, Prevotellaceae showed a positive correlation with both *Occludin* and *Claudin-1* expressions (p<0.01). It is worth noting that claudins play an essential role as structural components of tight junctions, while *Occludin*s are crucial for maintaining their stability and barrier function [53]. Furthermore, our findings indicated that oral administration of polyethylene microplastics (PE-MPs) had detrimental effects on intestinal barrier integrity in rats as evidenced by apparent histopathological alterations in the jejunum; notably decreased expressions of *Occludin* and *Claudin-1* were observed after PE-MP exposure [54]. Additionally, numerous studies have confirmed that microplastics can influence gut microbiota composition [55–58]. Therefore, our results suggest that investigating changes in gut microbial communities following oral administration of microplastics could provide valuable insights into understanding the regulatory network involving "microplastic-intestinal microbiota-intestinal barrier" for future research.

## 5. Conclusion

In summary, this study confirms that polystyrene microplastics (PS-MPs) disrupt the small intestine’s microstructure in mice, reduce the expression of tight junction genes in the duodenum and jejunum, and cause dysbiosis in the gut microbiota, thereby affecting intestinal barrier function. Exposure to PS-MPs also leads to significant differences in gut microbiota composition and relative abundance of metabolism-related functional genes between males and females. These findings suggest a notable sex difference in how PS-MPs impact mouse gut microbiota, potentially influenced by hormone levels, immune system variations, estrogen effects, and genetic disparities; however, further research is needed to substantiate these factors’ exact roles. This study provides a foundation for understanding MPs-induced gut toxicity but acknowledges that potential effects and hazards on mammals are still being explored. Therefore, additional research focusing on distribution patterns and mechanisms of action specific to mammals while considering possible sex differences is necessary.
